# Screening infection prevention policies for equity impacts

**DOI:** 10.1017/ash.2023.229

**Published:** 2023-09-29

**Authors:** Caitlin McGrath, Yasaman Fatemi, Therese Mirisola, Tanya Ferreira, Adrienne D’Alo, Victoria Konold, Alicia Tieder, Ashley Durkin, Matthew Kronman, Danielle Zerr

## Abstract

**Background:** Infection prevention teams utilize policies to guide practice; however, some policies may inadvertently uphold institutional racism and discrimination. Our institution utilizes an equity impact assessment tool during new policy creation or existing policy updates to identify, reduce, eliminate, and prevent inequities in care. **Methods:** We reviewed all 119 current institution-wide policy documents related to or managed by the infection prevention division at Seattle Children’s Hospital using an institutional equity impact assessment tool. The tool asks 6 open-ended questions to help policy owners identify potential inequities and to evaluate how marginalized groups may be affected. Each policy was assessed for its potential to create or sustain inequities for patients, families, or staff. Policies determined to have potential inequities were examined for any language to suggest that equity considerations had been incorporated into the existing policy. Initial policy review was performed by 2 infection prevention physicians, and disagreements were resolved by consensus. We defined the presence of equity considerations as any explicit mention of disparate impact of the policy on marginalized groups or mitigation of such effect. **Results:** Of the 119 policies reviewed, 43 (36%) were identified as having substantial potential to impact marginalized groups and create or sustain inequities. Among them, 42 (98%) of these policies lacked existing equity considerations. The policies with potential equity implications covered the following categories: COVID-19 (including masking, workforce restriction, testing), visitor restrictions, tuberculosis, central-line–associated bloodstream infections (CLABSIs), multidrug-resistant organisms (MDROs), public health reporting, medical behavioral unit policies, off-site affiliate housing policies, special pathogens program (including Ebola, MERS, SARS), surgical-site infections, home care including dialysis, and occupational health-related policies. Examples of policies that did not highlight inequities included those pertaining to construction, water intrusion, and transmission-based precautions. One example of change driven by use of the equity impact assessment tool concerned communication with patients and families about tuberculosis isolation and resulted in creation of a standardized multidisciplinary care conference to better communicate tuberculosis isolation processes (including testing required, visitor restrictions, and anticipated duration of isolation) to families in their language of care. **Conclusions:** Hospital-wide infection prevention policies have the potential to create or sustain existing inequities. Systematic consideration of equity implications using an equity impact assessment framework could be the first step in mitigating these effects and can result in concrete actions to reduce systemic racism.

**Disclosure:** None

